# Comparative genomics among cyst nematodes reveals distinct evolutionary histories among effector families and an irregular distribution of effector‐associated promoter motifs

**DOI:** 10.1111/mec.16505

**Published:** 2022-06-01

**Authors:** Joris J. M. van Steenbrugge, Sven van den Elsen, Martijn Holterman, Jose L. Lozano‐Torres, Vera Putker, Peter Thorpe, Aska Goverse, Mark G. Sterken, Geert Smant, Johannes Helder

**Affiliations:** ^1^ Laboratory of Nematology Wageningen University & Research Wageningen The Netherlands; ^2^ Solynta Wageningen The Netherlands; ^3^ School of Medicine, Medical & Biological Sciences University of St. Andrews St Andrews UK

**Keywords:** cyst nematodes, DOG box, *Globodera*, *Heterodera*, nematode effectors

## Abstract

Potato cyst nematodes (PCNs), an umbrella term used for two species, *Globodera pallida* and *G. rostochiensis*, belong worldwide to the most harmful pathogens of potato. Pathotype‐specific host plant resistances are essential for PCN control. However, the poor delineation of *G. pallida* pathotypes has hampered the efficient use of available host plant resistances. Long‐read sequencing technology allowed us to generate a new reference genome of *G. pallida* population D383 and, as compared to the current reference, the new genome assembly is 42 times less fragmented. For comparison of diversification patterns of six effector families between *G. pallida* and *G. rostochiensis*, an additional reference genome was generated for an outgroup, the beet cyst nematode *Heterodera schachtii* (IRS population)*.* Large evolutionary contrasts in effector family topologies were observed. While VAPs (venom allergen‐like proteins) diversified before the split between the three cyst nematode species, the families GLAND5 and GLAND13 only expanded in PCNs after their separation from the genus *Heterodera*. Although DNA motifs in the promoter regions thought to be involved in the orchestration of effector expression (“DOG boxes”) were present in all three cyst nematode species, their presence is not a necessity for dorsal gland‐produced effectors. Notably, DOG box dosage was only loosely correlated with the expression level of individual effector variants. Comparison of the *G. pallida* genome with those of two other cyst nematodes underlined the fundamental differences in evolutionary history between effector families. Resequencing of PCN populations with different virulence characteristics will allow for the linking of these characteristics to the composition of the effector repertoire as well as for the mapping of PCN diversification patterns resulting from extreme anthropogenic range expansion.

## 1 INTRODUCTION

Worldwide, affordable food and feed production depends on large‐scale monocropping. For practical and economic reasons crop homogeneity in terms of yield quality and quantity is essential. At the same time, such systems are intrinsically vulnerable to damage by pests and pathogens. The highest susceptibility to biotic stressors is found in genetically homogeneous crops. Potato is the third most important staple food (Birch et al., 2012), and in most production systems clonally propagated seed potatoes are used as starting material. Such production systems need rigorous disease management. Potato cyst nematodes (PCNs), the common name for two species, *Globodera pallida* and *G. rostochiensis*, are among the primary yield‐limiting potato pathogens worldwide. PCNs co‐evolved with potato in the Andes in South America (see, e.g., Plantard et al., 2008), and proliferation of potato as a main crop outside of its native range was unintentionally paralleled by an enormous range expansion of PCNs. For decades, PCN control has mainly been dependent on the application of nematicides. Due to the nonspecific nature of these nematicides, they have a highly negative impact on the environment, and their use is therefore either banned or severely restricted in most parts of the world. Currently, crop rotation and the use of resistant potato varieties are the main means for PCN control. For economic reasons, the use of plant resistances is preferred over crop rotation. However, potato resistance genes such as *H1* (Toxopeus & Huijsman, 1952), *Gro1‐4* (Paal et al., 2004), *Gpa2* (Bakker et al., 2003) and *H2* (Strachan et al., 2019) are only effective against specific pathotypes of one of these PCN species. Nevertheless, there is no robust (molecular) pathotyping scheme that would allow for matching of the genetic constitution of field populations with effective host plant resistance genes.

Effectors are proteins secreted by plant pathogens that allow manipulation of the physiology of the host plant and interfere with the host's innate immune response in favour of the invading organism (e.g., Stergiopoulos & De Wit, 2009). PCN effectors have some peculiar characteristics. With at least one known exception, HYPer variable proteins (HYPs) (Eves‐van den Akker et al., 2014), most effectors are produced in large single‐celled glands referred to as the subventral and dorsal oesophageal glands. These glands empty into the pharynx lumen, and the lumen is connected to a hollow protrusible stylet with which nematodes pierce plant cell walls. Via the orifice of the stylet, effector proteins are transferred to the apoplast or the cytoplasm of infected host plant cells. Notably, subventral gland effectors are functional during plant penetration. Subsequently, dorsal gland secretions are responsible for feeding site induction and suppression of the host's innate immune system (Smant et al., 2018). It has been hypothesized that common transcription factors and/or common promoter motifs might facilitate coordinated expression of effectors during the infection process. Such mechanisms have been identified to regulate effector expression in plant pathogenic fungi and oomycetes (Jones et al., 2019; Roy et al., 2013). Also, among plant‐parasitic nematodes, promotor motifs have been identified upstream of effectors that could contribute to the orchestration of the infection process. In the case of the PCN *G. rostochiensis*, a DOrsal Gland motif (“DOG box”) was identified by Eves‐van den Akker et al. (2016). For the pinewood nematode *Bursaphelenchus xylophilus*, a regulatory promotor motif referred to as STATAWAARS was demonstrated to affect effector expression (Espada et al., 2018). Expression of several effectors of Clade I tropical root‐knot nematodes (Tandingan De Ley et al., 2002) was suggested to be steered by a putative *cis*‐regulatory motif “Mel‐DOG” (*Meloidogyne* DOrsal Gland, Da Rocha et al., 2021).

Probably as a reflection of the co‐evolution between nematodes and their host(s), effectors are typically encoded by multigene families showing family‐specific levels of diversification (Masonbrink et al., 2019; Van Steenbrugge et al., 2021). Cyst nematodes harbour numerous effector families (see, e.g., Pogorelko et al., 2020), and genome (re‐)sequencing is a rigorous approach to generate comprehensive overviews of PCN effector family compositions. The first genomes of *G. pallida* and *G. rostochiensis* were published by Cotton et al. (2014) and Eves‐van den Akker et al. (2016). Although this constituted a major step forward, both genomes are highly fragmented, hampering effector family inventories. Recently, long‐read technology allowed for the generation of a less fragmented and more complete reference genome for *G. rostochiensis* with—among other things—a 24‐fold reduction in the number of scaffolds as compared to the initial reference genome (Van Steenbrugge et al., 2021). Here, we present a novel reference genome for the other PCN, *G. pallida*, characterized by a 42‐fold reduction in the number of scaffolds, together with a reference genome of the beet cyst nematode *Heterodera schachtii*. The *H. schachtii* genome was used to establish the polarity of effectorome contrasts between the two PCN species. Detailed knowledge of the nematode's effector repertoire, a complete overview of variants within effector families and insights in the evolutionary history of individual effector families are essential for a molecular pathotyping scheme. In addition to comparing effector diversification patterns, we investigated DOG box distribution and DOG box dosage (up to 16 DOG boxes were observed per putative promoter region) both within and among effector families. Scrutinizing putative effector promoter regions in three reference genomes allowed us to pinpoint the distribution of this putative regulatory motif among cyst nematode species, as well as among and within effector families. Subsequently, the impact of these new, long‐read technology‐based reference genomes on ecological PCN diversification in general and on the development of effectorome‐based pathotyping systems for PCNs in particular is discussed.

## 2 MATERIALS AND METHODS

### 2.1 DNA isolation and sequencing

Cysts from *Globodera pallida* line D383 were used as starting material for the collection of preparasitic second‐stage juveniles (J2). Pa2‐D383 is a relatively avirulent *G. pallida* population from The Netherlands (Rouppe van der Voort et al., 1998). J2s were concentrated, and sucrose centrifugation was used to purify the nematode suspension (Jenkins, 1964). After multiple rounds of washing the purified nematode suspension in 0.1 m NaCl, nematodes were resuspended in sterilized MQ water. Juveniles were lysed in a standard nematode lysis buffer with proteinase K and beta‐mercaptoethanol at 60°C for 1 hr as described in Holterman et al. (2006). The lysate was mixed with an equal volume of phenol/chloroform/isoamyl alcohol (25:24:1) (pH 8.0) following a standard DNA purification procedure, and finally, DNA was precipitated with isopropanol. After washing the DNA pellet with 70% ethanol several times, it was resuspended in 10 mm Tris–HCl (pH 8.0). *G. pallida* D383 DNA (10–20 mg) was sequenced using Pacific Biosciences SMRT sequencing technology at Bioscience (Wageningen Research). Additionally, *G. pallida* D383 DNA (10–20 mg) was sequenced using Illumina NovaSeq sequencing technology at Bioscience (Wageningen Research). DNA (30 mg) from *Heterodera schachtii* (IRS, a Dutch beet cyst nematode population) was isolated with a procedure similar to the one used for *G. pallida*, but DNA was precipitated using an ice‐cold ethanol precipitation step (Jain et al., 2018). DNA fragments of <10 kb were depleted using a short read eliminator kit (Circulomics SS‐100‐121‐01) and *H. schachtii* DNA (15 mg) was sequenced using Oxford Nanopore technology at NexOmics.

### 2.2 Genome assemblies and synteny

For *G. pallida* D383, raw PacBio reads, and for *H. schachtii* IRS, Oxford Nanopore reads were corrected to, in essence, merge haplotypes using the correction mode in canu (Koren et al., 2017), by reducing the error rate to a maximum of 15% and the corrected coverage to a minimum of 200. Using wtdgb2 version 2.3 (Ruan & Li, 2020), multiple initial genome assemblies were generated based on the corrected Nanopore reads while manually refining the parameters minimal read length, k‐mer size and minimal read depth. These parameters were optimized to generate an assembly close to the expected genome size of *G. pallida* and *H. schachtii*. After optimization, for *G. pallida*, a minimum read length cut‐off of 5000, minimal read depth of 6 and a k‐mer size of 18 were used. To generate the assembly of *H. schachtii*, a minimum read length cut‐off of 6000, minimal read depth of 8 and a k‐mer size of 21 were used. The remaining haplotigs were pruned from the assemblies using purge haplotigs version 1.0.4 (Roach et al., 2018). The contigs from the assemblies were then improved using finishersc version 2.1 (Lam et al., 2015) at default settings and scaffolded using sspace‐longread version 1.1 (Boetzer & Pirovano, 2014). Gaps in the assemblies were then filled using gapfiller version 1.0 (Van Steenbrugge, 2021). For *G. pallida*, the resulting assembly was polished with PacBio reads by three iterations of arrow version 2.3.3 (https://github.com/PacificBiosciences/GenomicConsensus), followed by five iterations of polishing with pilon version 1.23 (Walker et al., 2014) using PacBio and Illumina NovaSeq reads. Finally, the assembly of *H. schachtii* was polished with medaka version 1.4.1 model r941_prom_hac_g3210 using Nanopore sequencing reads, followed by five iterations of polishing with pilon using Illumina reads. Repeat regions were softmasked using repeatmodeler version 1.0.11 (https://github.com/Dfam‐consortium/RepeatModeler) and repeatmasker version 4.0.9 (Tarailo‐Graovac & Chen, 2009). Using braker version 2.1.2 (Brůna et al., 2021), gene annotations were predicted for both assemblies at default settings outputting gff3 annotations and aided by RNAseq data of different life stages (see section 2.3 for experimental set up; *G. pallida*: NCBI Bioproject PRJEB2896, *H. schachtii*: PRJNA767548). Full details on the generation of the genome assemblies and prediction of genes are available at https://github.com/Jorisvansteenbrugge/Gros_Gpal_Hsch. For *Globodera rostochiensis*, the Gr‐Line19 genome assembly described in Van Steenbrugge et al. (2021) was used (NCBI GenBank assembly accession: GCA_018350325.1).

The synteny between the *G. rostochiensis*, *G. pallida* and *H. schachtii* genomes was assessed by a progressive genome alignment using mauve version 2.4.0 (Darling et al., 2004). The resulting alignments of regions larger than 1 kb and larger than 3 kb were visualized in circos version 0.69–9 (Krzywinski et al., 2009).

Completeness of the assemblies was assessed using busco version 5.2.2 using the eukaryota_odb10 database (Manni et al., 2021). busco was run in genome mode using metaeuk version 5.34c21f2 as the gene predictor (Levy Karin et al., 2020).

### 2.3 Generation of RNAseq data from various life stages

Stem cuttings of potato genotype SH (Bakker et al., 2003) were grown in vitro and inoculated with infective second‐stage juveniles of *G. pallida* population D383. Plants were kept at 18°C in the dark and nematode‐infected potato root segments were collected 3 and 6 days after infection (three biological replicates). Samples were collected and snap‐frozen in liquid nitrogen. RNA extraction was performed using the Maxwell 16 LEV‐plant RNA kit (Promega) following the manufacturer's protocol. RNA degradation and contamination were monitored on a 1% agarose gel. Purification was checked by using a NanoPhotometer spectrophotometer (IMPLEN). RNA integrity and quantification were assessed by using the RNA Nano 6000 Assay Kit of the Bioanalyzer 2100 system (Agilent Technologies). RNA sequencing was done at Novogene by using a Novaseq 5000 PE150 platform, providing at least 50 million clean paired‐end reads of 150 bp per sample. See section 2.2 for data accessibility.

### 2.4 Identification of effector homologues

Effector gene families were identified in the genomes based on the predicted genes by braker2. Homologues for Gr‐1106/Hg‐GLAND4 were identified using hmmsearch (hmmer version 3) with a hidden Markoc model (HMM) profile that was produced with hmmbuild based on GenBank entries JQ912480 to JQ912513. SPRY homologues were identified with hmmer using a precalculated profile HMM in the PFAM database (PF00622). Homologues to CLE‐like proteins were identified with a profile HMM‐\ produced with hmmbuild based on UniProt sequences (D1FNJ7, D1FNJ9, D1FNK0, D1FNK2, D1FNK3, D1FNK4, D1FNK5, D1FNK8). Homologues to venom allergen‐like proteins (VAPs) were identified with a profile HMM‐based produced with hmmbuild using Uniprot sequences (Q8MQ79, A0A0K3AST9, P90958, Q19348, A0A0K3AWG2, Q967G4, Q9BID5, A0A3Q8UEU8, Q963I7, B8LF85, A7X975, A0A7G7LJV8). For HMM‐based searches, an E‐value threshold of 0.0001 was used. Hg‐GLAND5 and Hg‐GLAND13 were identified with blastp searches with GenBank sequences KJ825716 and KJ825724, respectively, maintaining thresholds at 35% identity, 50% query coverage and an E‐value of 0.0001. Each effector homologue was tested for the presence of a signal peptide for secretion by phobius version 1.01 (Käll et al., 2007) and the presence of one or multiple DOG box motifs in the promoter region using a custom script (available on GitHub https://github.com/Jorisvansteenbrugge/Gros_Gpal_Hsch).

For *G. rostochiensis*, effector annotations were used as described in Van Steenbrugge et al. (2021), except for CLE‐like proteins. CLE variant Gros19_g16105.t1 was excluded because the gene model probably contains errors, and the exact location of this variant in the phylogenetic tree is therefore uncertain. Furthermore, the HMM scoring cut‐off was lowered to 300 to include two additional potential CLE variants.

### 2.5 Phylogeny

A multiple sequence alignment was generated for each effector gene family using muscle version 3.8.1551 based on gene coding sequences to infer the phylogeny of effector gene families between species. Next, phylogenetic trees were produced with raxml version 8.2.12 (Stamatakis, 2014) using the GTRGAMMA model with 100 bootstrap replicates. The GTRGAMMA model was selected based on recommendations by modeltest‐ng version 0.2.0 (Darriba et al., 2020). Finally, using figtree version 1.4.4, the resulting trees were visualized and annotated.

### 2.6 DOGbox identification and gene expression analysis

RNAseq reads (BioSample SAMN22835760) were mapped to the *G. pallida* D383 genome, reads (Bio‐Sample SAMN22835918) were mapped to the *G. rostochiensis* Gr‐Line19 genome and reads (Bio‐Sample SAMN22818360) were mapped to the *H. schachtii* IRS genome using hisat2 version 2.1.0, generating alignments tailored for transcript assemblers (‐‐dta option). Based on the alignments, abundances were estimated and normalized to transcripts per million (TPM) for the transcripts predicted by braker2 using stringtie version 2.1.7b (Kovaka et al., 2019). Expression values of SPRYSEC genes in all three cyst nematode species were extracted, and plotted against the number of DOG box motifs in each gene. Spearman's rank‐order correlation was used to determine the relationship between TPM and the number of DOG box motifs.

## 3 RESULTS

### 3.1 Use of long read sequence technologies to generate novel reference genomes

The mapping of diversification patterns of effector families requires a high‐quality reference genome with preferably a low number of scaffolds and a minimal gap length. Cotton et al. (2014) were the first to present a reference genome of the PCN species *Globodera pallida*. For our specific purpose (i.e., the generation of complete inventories of effector families), this reference genome was too fragmented, and the total gap length was too large. PacBio long‐read technology allowed us to generate a new reference genome from the *G. pallida* population D383 with a 42‐fold reduction of scaffolds and a 21‐fold reduction of the total gap length. As one consequence, the number of predicted genes increased from 16,403 to 18,813, and the level of completeness as estimated by busco increased by more than 10% (Table 1).

**TABLE 1:** Comparative genome statistics of four cyst nematode genome assemblies. In bold, data from the current paper; data on Globodera pallida Lindley and G. rostochiensis Line 19 genomes were published by respectively Cotton et al. (2014) and Van Steenbrugge et al. (2021)

Nematode species population	Size (Mb)	Number of scaffolds	N50 (Mb)	N90 (Mb)	Number of gaps	Gap length	Number of genes	Number of transcripts
*Globodera pallida* Lindley	124.7	6873	0.122	0.011	6873	19,990,795	16,403	16,403
*G. pallida* Pa2‐D383	**113**	**163**	**2**.**9**	**0**.**515**	**22**,**788**	**945**,**137**	**18**,**813**	**27**,**787**
*G. rostochiensis* Line 19	92	173	1.70	0.582	2733	130,000	17,928	21,037
*Heterodera schachtii* IRS	**190**	**705**	**0**.**5**	**0**.**132**	**705**	**4**,**285**,**731**	**29**,**851**	**31**,**564**
BUSCO								
*G. pallida*, Lindley	C:49.0%:[S:44.3%,D:4.7%],F:21.6%,M:29.4%,n:255							
*G. pallida*, D383	C:59.2%[S:58.4%,D:0.8%], F:19.6%,M:21.1%,n:255							
*G. rostochiensis* Line 19	C:63.5%[S:62.7%,D:0.8%],F:17.6%,M:18.9%,n:255							
*H. schachtii*, IRS	C:86.3% [S:80.8%, D:5.5%], F:7.1%, M:6.6%, n:255							

In addition, we assembled the genome sequence of the IRS population of the beet cyst nematode *Heterodera schachtii*. This allowed us to compare effector family diversification among the two PCN species, *G. pallida* and *G. rostochiensis*, and establish the polarity of these contrasts by using *H. schachtii* as an outgroup (both *Globodera* and *Heterodera* belong to the family Heteroderidae). The current genome size, 190 Mb, is slightly above the genome size estimated by flow cytometry, 160–170 Mb (Siddique et al., 2021). Notably, both the predicted number of genes and transcripts were about 50% higher in *H. schachtii* than in the two *Globodera* species (Table 1). These figures largely correspond to a *H. schachtii* genome assembly that was recently published from a German population referred to as “Bonn” with an assembly size of 179 Mb, and 26,700 predicted genes (Siddique et al., 2021).

**FIGURE 1:**
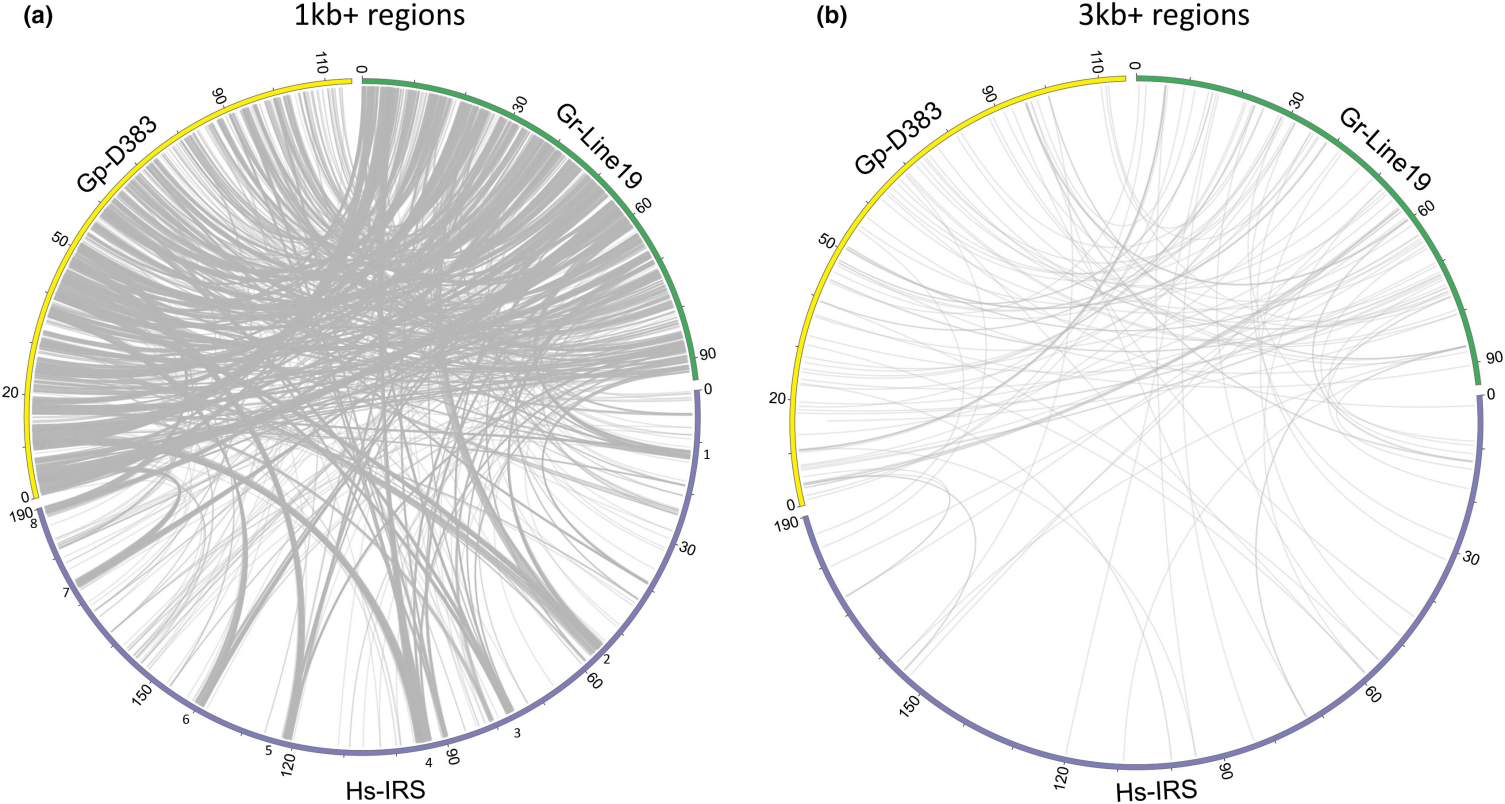
Synteny between *Globodera pallida* (population D383), *Globodera rostochiensis* (Gr‐Line19) and *Heterodera schachtii* (population IRS) based on a progressive genome alignment in mauve. (a) Syntenic regions larger than 1 kb, (b) syntenic regions larger than 3 kb. In (a), *H. schachtii* genome regions are indicated where multiple syntenic regions cluster together into segments spanning over 2 Mb (segments 1–8). Note that these segments have equivalents in both *G. pallida* and *G. rostochiensis*

Two synteny plots were generated based on the alignment of regions >1 and >3 kb to compare the genomic organization of the three cyst nematode species. Not unexpectedly, the two PCN species share numerous >1‐kb regions (Figure 1a). In the *H. schachtii* genome, several homologous >1‐kb regions cluster together in genomic segments that span over 2 Mb (Figure 1a, segments 1–8). The homologous >1‐kb regions in these segments have equivalents in both *G. pallida* and *G. rostochiensis*. Alignment of >3‐kb fragments severely reduced the number of homologous regions among the three cyst nematode species (Figure 1b). Nevertheless, the number of shared >3‐kb regions between *G. pallida* and *G. rostochiensis* (*N* = 76) is considerably higher than the number of shared regions between *H. schachtii* and *G. rostochiensis* (*N* = 23) (Figure 1b).

### 3.2 Effector family selection

In our comparison between the three cyst nematode species, we concentrated on effectors. Cyst nematodes were shown to harbour numerous effector families (Pogorelko et al., 2020). Here we concentrated on six effector families. For four of these families, one or more representatives are known to be involved in the suppression of plant innate immune system: SPRYSEC (Diaz‐Granados et al., 2016; Mei et al., 2018), GLAND4 (also referred to as Gr‐1106) (Barnes et al., 2018), GLAND5 (also referred to as G11A06) (Yang, Pan, et al., 2019) and VAP (Wilbers et al., 2018). CLE (Wang et al., 2021) is an intriguing effector family involved in feeding site induction, and the GLAND13 (Danchin et al., 2016) family is essential in the hydrolysis of plant sugars once they are taken up by the nematode.

### 3.3 SPRYSECs

**FIGURE 2:**
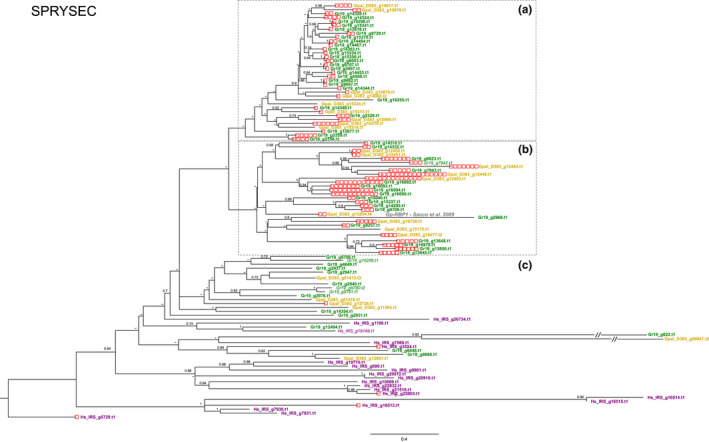
Phylogeny of SPRYSEC effector genes (see, e.g., Diaz‐Granados et al. (2016) of *Globodera pallida* (population D383) (ochre), *Globodera rostochiensis* (Gr‐Line19) (green) and *Heterodera schachtii* (population IRS) (purple). A multiple sequence alignment was made using muscle on the coding sequence. A phylogenetic tree was made using raxml using a GTRGAMMA model, validated by 100 bootstrap replicates. Bootstrap values <50% are indicated by a dash. GenBank IDs in italics in lighter shades of ochre, green or purple are used to indicate effector variants with at least one predicted transmembrane domain. Boxed clusters (A) and (B) highlight two moderately well‐supported subclades with on average moderate (A) and high DOG box dosages. (C) is the basal part of the SPRYSEC tree

SPRYSEC is an acronym for a family of secreted effectors with an SP1a/RYanodine receptor domain. This family was recently shown to be highly diverged in the PCN *G. rostochiensis* (van Steenbrugge et al., 2021). Figure 2 shows a phylogenetic tree with (supposedly) all SPRYSECs present in the three cyst nematode species under investigation. The number of paralogues in *G. pallida*, *G. rostochiensis* and *H. schachtii* is respectively 24, 60 and 20. Despite the poor backbone resolution of the SPRYSEC tree, two moderately supported SPRYSEC clades (A and B) could be distinguished. Clade A comprises SPRYSEC variants exclusively from the two PCN species, and *G. pallida* SPRYSEC paralogs are interspersed with *G. rostochiensis* SPRYSEC variants. SPRYSEC Clade A is characterized by zero to six DOG box elements. Clade B harbours fewer SPRYSEC paralogues than Clade A (27 vs. 35 in Clade A). Notably, Gr19_g7942 includes a transmembrane domain (and is therefore unlikely to be secreted), whereas three DOG box elements are present in the promoter region directly upstream of this paralogue. Clade B is characterized by a mix of SPRYSEC variants solely originating from *G. pallida* and *G. rostochiensis*. Compared to Clade A, Clade B is typified by an overall higher DOG box dosage (on average, 1.7 and 5.2 DOG boxes per paralogue). Up to 16 DOG box elements were identified in the promoter regions of paralogues in Clade B. The more basal part of the SPRYSEC tree (Figure 2, part c) harbours, next to paralogues from the two PCN species, all 20 SPRYSEC variants from *H. schachtii*. Five promoter regions of the 35 SPRYSEC paralogues in this part of the SPRYSEC tree harbour a single DOG box. Both *H. schachtii* and *G. rostochiensis* harbour SPRYSEC paralogues with at least one transmembrane domain (gene ID in italics with lighter colour).

Four *G. pallida* SPRYSEC proteins variants referred to as RBP‐1‐D383‐1, −2, −3 and −4 that were recognized by the potato resistance gene *Gpa2* (Sacco et al., 2009) correspond to Gpal_D383_g12854 (Figure 2). The four marginally distinct RBP‐1 s presumably include allelic variants of Gpal_D383_g12854. Notably, the closest equivalent of *Gp*SPRY‐414‐2, a SPRYSEC isolated from the *G. pallida* Pa2/3 population “Lindley” (Mei et al., 2018), was not preceded by a signal peptide for secretion in our genome assembly, and therefore could not be included in Figure 2.

### 3.4 GLAND4

**FIGURE 3:**
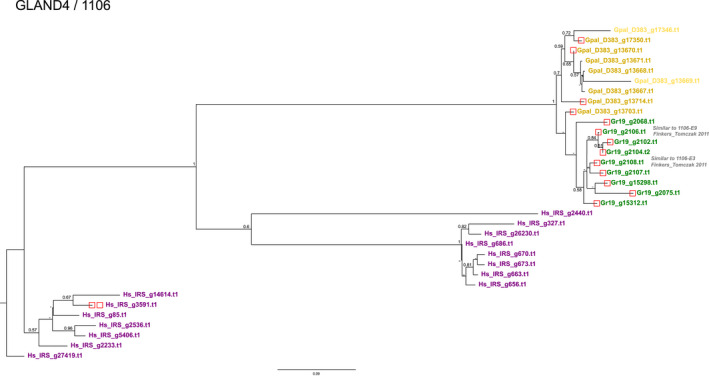
Phylogeny of GLAND 4 (equivalent to 1106, see, e.g., Noon et al., 2015) effector genes of *Globodera pallida* (population D383) (ochre), *Globodera rostochiensis* (Gr‐Line19) (green) and *Heterodera schachtii* (population IRS) (purple). A multiple sequence alignment was made using muscle on the coding sequence. A phylogenetic tree was made using raxml using a GTRGAMMA model, validated by 100 bootstrap replicates. Bootstrap values <50% are indicated by a dash. GenBank IDs in lighter shades of ochre, green or purple are used to indicate effector variants lacking a signal peptide for secretion

The number of GLAND4 (also referred to as Gr‐1106) paralogues in Gr‐Line19, Gp‐D383 and Hs‐IRS is respectively 10, nine and 15. The phylogenetic analysis yields a tree with a well‐supported backbone (Figure 3) showing a clear separation between the outgroup *H. schachtii* and both *Globodera* species. Except for Gpal_D383_g13703, which is positioned at the base of the *G. rostochiensis* cluster, GLAND4 variants end up in separate species‐specific clusters. On the other hand, Hs‐IRS paralogues show more intraspecific diversification. All but two *G. pallida* paralogues (Gpal_D383_g17346.t1 and Gpal_D383_g13669) contain a signal peptide for secretion. For all but one of the GLAND4 genes in Gr‐Line19, the promoter region included a DOG box motif, while promoter regions of only four GLAND4 genes in Gp‐D383 and one in Hs‐IRS contained such a motif.

Previously, overexpression of a subset of *G. rostochiensis* GLAND4 (1106) variants in potato resulted in enhanced host plant susceptibility as observed by a significant increase in the number of cysts formed (Finkers‐Tomczak, 2011). The equivalents of two of these variants, E3 and E9, in *G. rostochiensis* line 19 are indicated in Figure 3.

### 3.5 GLAND5

**FIGURE 4:**
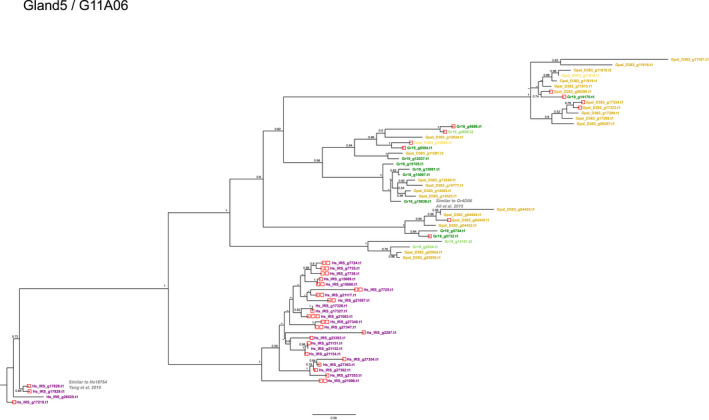
Phylogeny of GLAND 5 (equivalent to G11A06, see, e.g., Noon et al., 2015) effector genes of *Globodera pallida* (population D383) (ochre), *Globodera rostochiensis* (Gr‐Line19) (green) and *Heterodera schachtii* (population IRS) (purple). A multiple sequence alignment was made using muscle on the coding sequence. A phylogenetic tree was made using raxml using a GTRGAMMA model, validated by 100 bootstrap replicates. Bootstrap values <50% are indicated by a dash. GenBank IDs in lighter shades of ochre, green or purple are used to indicate effector variants lacking a signal peptide for secretion

With 13 homologues, the GLAND5 effector family (also referred to as G11A06) was significantly less diversified in Gr‐Line19 than in Gp‐D383 and Hs‐IRS with respectively 25 and 27 paralogues. In all three species, the majority of the GLAND5 paralogues harbour a signal peptide for secretion. Note that a relatively high percentage of the GLAND5 paralogues in Gr‐Line19 was not preceded by a signal peptide for secretion (23%). In contrast, in *H. schachtii* and *G. pallida*, respectively, 88.9% and 92% of the paralogues comprised a signal peptide. Phylogenetic analysis (Figure 4) shows that GLAND5 is a diversified gene family. Several well‐supported branching events define a set of subclades that either exclusively comprises *H. schachtii* or contain GLAND5 variants from both PCN species in the more distal branches. Even though the GLAND5 paralogues Gr‐Line19 and Gp‐D383 occur together in individual subclades, no obvious sets of potential orthologues between the two species could be identified. In *H. schachtii*, 82% of the paralogues contain at least one DOG box motif in the promoter region. Out of the three GLAND5 paralogues without a signal peptide for secretion, two (Hs‐IRS_g6495.t1 and Hs‐IRS_g22438.t1) had at least one DOG box motif in their promoter region. DOG boxes were less prominently present among the *G. rostochiensis* and *G. pallida* GLAND5 variants (39% and 20% of the paralogues).

One of the *G. rostochiensis* paralogues (Gr19_g15036) showed similarity with *Gr*4D06, a GLAND6 effector protein described by Ali et al. (2015) (Figure 4). This effector has an unknown function, and expression *in planta* did not result in a phenotype. GLAND5 and GLAND6 are related effector families (Noon et al., 2015). *H. schachtii* GLAND5 variant Hs_IRS_g17828 showed similarity to *Hs18764* described by Yang, Dai, et al. (2019), and overexpression of *Hs18764 in Arabidopsis thaliana* resulted in an increased susceptibility to *H. schachtii*.

### 3.6 VAPs (venom allergen‐like proteins)

**FIGURE 5:**
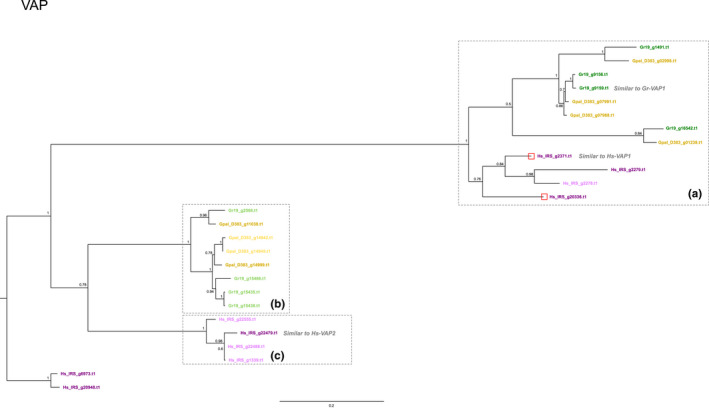
Phylogeny of VAP (venom allergen‐like protein, see, e.g., Wilbers et al., 2018) effector genes of *Globodera pallida* (population D383) (ochre), *Globodera rostochiensis* (Gr‐Line19) (green) and *Heterodera schachtii* (population IRS) (purple). A multiple sequence alignment was made using muscle on the coding sequence. A phylogenetic tree was made using raxml using a GTRGAMMA model, validated by 100 bootstrap replicates. Bootstrap values <50% are indicated by a dash. GenBank IDs in lighter shades of ochre, green or purple are used to indicate effector variants lacking a signal peptide for secretion

**FIGURE 6:**
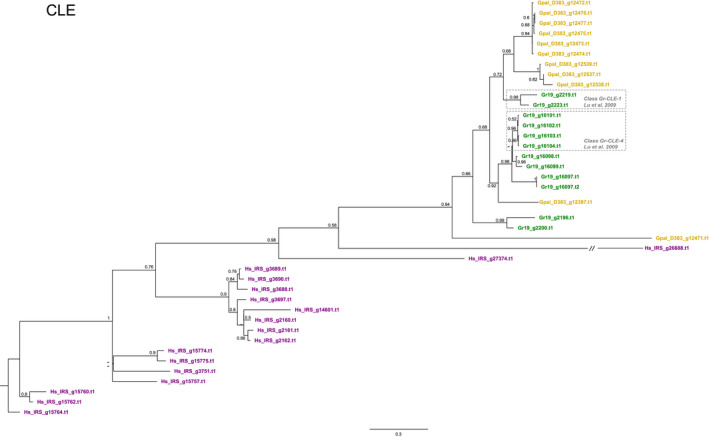
Phylogeny of CLE (CLAVATA3/ESR‐related peptides, see, e.g., Lu et al., 2009) effector genes of *Globodera pallida* (population D383) (ochre), *Globodera rostochiensis* (Gr‐Line19) (green) and *Heterodera schachtii* (population IRS) (purple). A multiple sequence alignment was made using muscle on the coding sequence. A phylogenetic tree was made using raxml using a GTRGAMMA model, validated by 100 bootstrap replicates. Bootstrap values <50% are indicated by a dash. GenBank IDs in lighter shades of ochre, green or purple are used to indicate effector variants lacking a signal peptide for secretion

The levels of diversification in the VAP effector family were highly comparable between the three cyst nematode species. In Gp‐D383, Hs‐IRS and Gr‐Line19, respectively, eight, eight and 10 VAP paralogues were identified. Phylogenetic analysis resulted in a tree with a well‐supported backbone (Figure 5). The tree contains three clusters (Figure 5, boxes A, B and C) with a high level of diversification between the clusters. At the base of the tree, a small cluster of four *H. schachtii* paralogues (Figure 6, box C) is present that all lack a signal peptide for secretion. Box B harbours Gp‐D383 and Gr‐Line19 VAP paralogues, of which all but two lack a signal peptide for secretion. Two subclusters are present in Box B: one with exclusively Gr‐Line19 variants, a second one with just Gp‐D383 variants, and the third with an orthologous pair between Gr‐Line19 and Gp‐D383. In the largest cluster at the top of the tree (Figure 5, box A), VAP paralogues of all three species are present, including the only two secreted VAP variants of Hs‐IRS with a DOG box motif in the promoter region.

Heterologous expression of three VAPs from *G. rostochiensis* (Gr‐VAP1) and *H.schachtii* (Hs‐VAP1, 2) resulted in the loss of basal immunity to the corresponding cyst nematode species as well as to some other, unrelated pathogens. It was concluded that these VAP variants interfere with the basal immune response of their host plants (Lozano‐Torres et al., 2014). VAP variants highly similar to Gr‐VAP1 and Hs‐VAP1, 2 are indicated in Figure 5.

### 3.7 CLE (CLAVATA3/ESR‐related peptides)

With 16 variants, the CLE‐like effector family is considerably more diversified in *H. schachtii* than in *G. pallida* and *G. rostochiensis* (respectively, 10 and 11 paralogues). Analysis of the CLE families on the three cyst nematode species resulted in a phylogenetic tree with a reasonably well‐resolved backbone (Figure 6). Note that nearly all variants are united in species‐specific clusters, and in this sense, the CLE diversification patterns resemble the patterns observed for the GLAND4 (Gr‐1106) family (Figure 3). Whereas Gp‐D383 and Gr‐Line19 are characterized by similar‐sized, moderately diverged clusters of CLE paralogues, the CLE family is far more diverged in *H. schachtii*.

In *G. rostochiensis*, two functional classes of CLE peptides have been described, CLE‐1 and CLE‐4 (Lu et al., 2009). The CLE‐1 class (Figure 6) comprises two Gr‐Line19 paralogues that show only distant homology to *G. pallida* CLEs. Similarly, four *G. rostochiensis* CLEs belonging to functional CLE class 4 (Figure 6) do not have clear equivalents in *G. pallida* and *H. schachtii*. Unlike all other effector families investigated so far, all CLE variants from the three cyst nematode species are preceded by a signal peptide for secretion. At the same time, none of them has a DOG box motif in the promoter region.

### 3.8 GLAND13 (glycosyl hydrolase family 32 [GH32] members)

**FIGURE 7:**
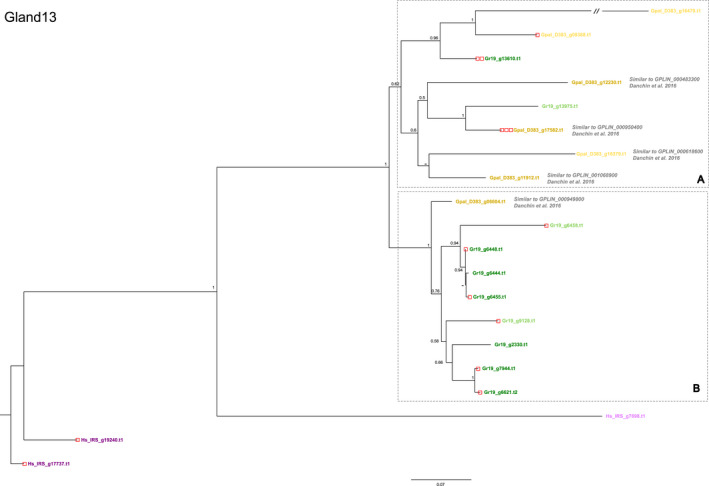
Phylogeny of GLAND13 (invertases, see, e.g., Danchin et al., 2016) effector genes of *Globodera pallida* (population D383) (ochre), *Globodera rostochiensis* (Gr‐Line19) (green) and *Heterodera schachtii* (population IRS) (purple). A multiple sequence alignment was made using muscle on the coding sequence. A phylogenetic tree was made using raxml using a GTRGAMMA model, validated by 100 bootstrap replicates. Bootstrap values <50% are indicated by a dash. GenBank IDs in lighter shades of ochre, green or purple are used to indicate effector variants lacking a signal peptide for secretion

GLAND13 effectors investigated so far have been identified in *G. pallida* and coded for invertases belonging to glycosyl hydrolase family 32 (GH32). While these enzymes are not secreted into the plant, they are essential as they catalyse the hydrolysis of the primary type of sugar the nematode takes up from its host, sucrose (Danchin et al., 2016). This gene family shows a large difference in the number of paralogues present in the three species; while Gr‐Line19 and Gp‐D383 harbour 10 and seven paralogues, Hs‐IRS holds only three copies. In the phylogenetic tree (Figure 7), the paralogues in *H. schachtii* are positioned at the tree's base. In Box A, paralogues of Gr‐Line19 and Gp‐D383 are interspersed, while in Box B, all paralogues except one are from Gr‐Line19. In Gr‐Line19, 70% of the GLAND13 paralogues comprise a signal peptide for secretion; slightly lower percentages (67% and 57%) were observed in Hs‐IRS and Gp‐D383. Variants showing high similarity to each of the five GLAND13 paralogues from *G. pallida* (population Lindley; indicated as GPLIN_*number*) are indicated in Figure 7.

For more than half of the GLAND13 effector variants in Gr‐Line19, a DOG box motif in the promoter region was shown. One gene, Gr19_g13610, contained the motif twice. In *H. schachtii*, this motif was present in two of three genes, while in *G. pallida*, DOG boxes were found in one variant with a signal peptide for secretion (Gpal_D383_g17582), and in one paralogue without such a signal (Gpal_D383_g09388). Note that these DOG box motifs were found in promoter regions of *G. pallida* effectors that are not expressed in the dorsal gland (Danchin et al., 2016). GLAND13 variants similar to the five invertase variants characterized by Danchin et al. (2016) including those with the highest expression levels (GPLIN_000950400 and GPLIN_001068900) are shown in Figure 7.

### 3.9 Effects of DOG box dosage on SPRYSEC expression

**FIGURE 8:**
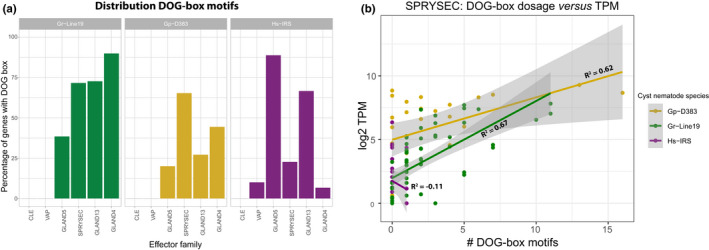
This distribution of a DOrsal Gland promoter element motif (“DOG box, Eves‐van den Akker et al., 2016) among a selection of cyst nematode species. (a) Percentages of variants per effector family with one or more a DOG boxes in their promoter region. (b) The relationship between DOG box dosage and expression level (expressed as log_2_ TPM [transcript count per million]). The relationship is expressed as a Spearman rank‐order correlation (*R* ^2^) per species, for the SPRYSEC gene family

Although DOG box motifs in the promoter regions of effector variants are present in many effector families, their presence is not a necessity for the functioning of an effector family. For example, none of the variants of the CLE family contained DOG box motifs, regardless of the species (Figure 6). Dorsal gland‐expressed effectors can therefore be expressed and secreted without the presence of DOG box motifs. This is further illustrated in Figure 8a, which shows DOG box distribution of over the six effector families. For the three cyst nematode species under investigation, it demonstrates that DOG boxes can be entirely absent (CLE), present in some species only (VAP) or present in all species (SPRYSEC, GLAND4, GLAND5, GLAND13). The ample presence of DOG boxes in the diversified SPRYSEC family among the two PCN species prompted us to investigate whether there is a correlation between the DOG box dosage and SPRYSEC expression levels. Although DOG boxes are present in the putative promoter regions of SPRYSECs from *H. schachtii*, they are relatively rare, and only single motifs were found. In Figure 8b, SPRYSEC effectors from all three cyst nematode species were taken into account. A modest correlation in *G. rostochiensis* and *G. pallida* (*R* ^2^ = .67 and .62 respectively) between the DOG box dosage and expression levels based on RNA abundance is present for this family. In *G. pallida*, in particular, high expression levels of SPRYSEC variants can be reached in the absence of DOG boxes in its promoter region (Figure 8b). For *H. schachtii*, a slightly negative correlation was found (*R* ^2^ = −.11) between DOG box dosage and expression levels.

## 4 DISCUSSION

In our attempt to fundamentally understand the interaction between plant‐parasitic nematodes and their hosts, the usefulness of high‐quality reference genomes of these pathogens is vital. Given the enormous impact of PCNs in all major potato production regions of the world, it is not surprising that high priority was given to the sequencing of both the *Globodera pallida* (Cotton et al., 2014) and the *Globodera rostochiensis* (Eves‐van den Akker et al., 2016) genome. This was done before long‐read sequencing technologies became available. Although some research questions can be well addressed with these reference genomes, less fragmented genomes are needed for studying effector diversification. Therefore, a new reference genome was generated from *G. pallida* population D383. As compared to the *G. pallida* Lindley genome assembly, this resulted in a 42‐fold reduction in the number of scaffolds and a 24‐fold increase in N50. In the comparison of the effectoromes of the two PCN species, we included a newly generated genome of the *Heterodera schachtii* population IRS as an outgroup. Note that reference genomes from these obligatory sexually reproducing pathogens are actually population‐based consensus genomes. Long read sequencing technologies require DNA from tens of thousands of genetically nonidentical nematodes. While an individual of these diploid species could theoretically carry a maximum of two haplotypes per locus, a population has the potential to carry many more. It is essential to mine these haplotypes and assemble them into a single haploid assembly to generate a proper reference. This is not a trivial process and requires specialized bioinformatics software (Roach et al., 2018). As the sizes of the current genome assemblies are comparable to the genome sizes assessed by flow cytometry, and as the busco duplication scores are relatively low, we assume that the current genome assemblies are a reasonable reflection of their actual constitution.

### 4.1 Effector diversification

In our analyses we concentrated on six selected effector families, and this selection included relatively widespread effector families such as CLE, GLAND13 and VAP, as well as families that appear to be cyst nematode lineage‐specific such as SPRYSEC, GLAND4 and GLAND5. Although the protein architecture is distinct between lineages (see, e.g., Mitchum et al., 2012), the CLE family—a category of effectors involved in feeding site induction—were shown to be present also in root‐knot and reniform nematodes (Rutter et al., 2014; Wubben et al., 2015). GLAND13 effectors, members of the glycosyl hydrolase family 32, were shown to be present in a range of root‐knot and cyst nematodes species as well as in other plant‐parasitic nematodes such as *Nacobbus aberrans* and *Rotylenchus reniformis* (Danchin et al., 2016). The distribution of VAPs within the phylum Nematoda is even broader (Wilbers et al., 2018). VAPs were discovered in the animal parasite *Ancylostoma caninum* (Hawdon et al., 1996). They were later isolated from the root‐knot nematode *Meloidogyne incognita* (Ding et al., 2000), and subsequently in a wide range of obligatory plant‐parasitic nematodes including various cyst nematode species. A number of VAP variants were shown to be implicated in the suppression of both PAMP‐ and effector‐triggered immunity (e.g., Li et al., 2021) for the burrowing nematode *Radopholus similis*. Our effector family selection also included families that (so far) appear to be specific to the cyst nematode lineages. These include SPRYSEC (Diaz‐Granados et al., 2016), GLAND4 (also referred to as Gr‐1106) and GLAND5 (also referred to as G11A06). For all of these effector families, at least a subset was shown to be involved in repression of the host plant immune system.

While comparing the overall diversification patterns of the six effector families under investigation, striking differences were observed. In SPRYSEC, GLAND5 (G11A06) and GLAND13 (GH32 members), virtually all *H. schachtii* paralogues appeared to be phylogenetically separate from the *G. pallida* and *G. rostochiensis* effector family variants, while representatives from the two PCN species were present in mixed clusters. These results should be taken with some caution as the backbone resolution of these phylogenetic trees ranges from poor (SPRYSEC) to robust (GLAND5, GLAND13). These patterns suggest that SPRYSEC, GLAND5 and GLAND13 effectors started to diversify after the split between *Heterodera* and *Globodera*.

Effector families GLAND4 (Gr‐1106) and CLE showed distinct diversification patterns; by far most paralogues are grouped in species‐specific clusters. As both effector families show a reasonable backbone resolution, we hypothesize that these effector families might have diverged after the split between *G. pallida* and *G. rostochiensis*.

Phylogenetic analysis of the VAP effector family in the three cyst nematode species revealed an opposite pattern, with almost complete mixtures of representative paralogues from the individual species. VAPs constitute an exceptionally widespread effector family within the phylum Nematoda (Wilbers et al., 2018), and our results indicate diversification of this family before the split between the cyst nematode genera *Globodera* and *Heterodera*.

### 4.2 Regulation of effector gene expression

Various stages of the parasitic life cycle of cyst nematodes such as plant invasion, feeding site induction and feeding site maintenance require the carefully orchestrated expression of distinct blends of effector proteins (Elashry et al., 2020; Thorpe et al., 2014). For some obligatory plant‐parasitic nematodes, promoter elements have been identified that were suggested to be involved in this orchestration (Da Rocha et al., 2021; Eves‐van den Akker et al., 2016). For the three cyst nematode species we showed the presence of a short DNA box motif (“DOG box”; ATGCCA) in the promoter region of some members of some of the effector families. The absence of DOG boxes in the CLE family, the scattered presence of DOG boxes in the other five families and the loose correlation between DOG box dosage and expression level lead us to conclude that DOG boxes might contribute to the orchestration of effector expression, but we see little evidence for a central role of this DNA motif in this process. Further investigation is necessary to elucidate the function of DOG boxes in effector regulation.

In plant‐pathogenic fungi, a few transcription factors have been identified that were shown to steer effector expression. SIX Gene Expression 1 (Sge1), a conserved member of the Gti1/Pac2 protein family, was instrumental in the regulation of effector repression in a range of fungal pathogens including *Verticillium dahlia* (Santhanam & Thomma, 2013), *Zymoseptoria tritici* (Mirzadi Gohari et al., 2014) and *Fusarium oxysporum* f. sp. *cubense* (Hou et al., 2018). Another example is AbPf2, a zinc cluster transcription factor from the necrotrophic plant pathogen *Alternaria brassicicola*. Via a loss of function approach, this transcription factor was shown to regulate the expression of eight putative effectors (Cho et al., 2013). Evidently, plant pathogenic fungi are only very distantly related to plant parasitic nematodes, and these examples should only be considered as an illustration of how effector expression is organized in other plant–pathogen systems.

## 5 CONCLUSIONS

The PCN *Globodera pallida* and its sibling species *G. rostochiensis* co‐evolved with potato in the Andes in South America. These pathogens have been introduced unintentionally in all major potato‐growing regions in the world. Currently, PCNs are the most harmful pathogens in potato production systems, and as a result of this extreme anthropogenic range expansion potatoes worldwide cannot be grown without adequate PCN management. For both *G. pallida* and *G. rostochiensis*, host plant resistance are the best way to control these soil pathogens. However, their effectiveness depends on proper matching between the genetic constitution of the PCN field population and the set of host pant resistances present in modern potato varieties. Niere et al. (2014) reported *G. pallida* populations that could no longer be controlled by any of the currently used potato cultivars. This, combined with inherent imperfections of the current *G. pallida* pathotyping system (e.g., Phillips & Trudgill, 1983), underlines the need for a new pathotyping system. Such a system will be based on distinctive effector variations present in any given PCN population. The availability of a high‐quality reference genome is a prerequisite for the development of such a system. We have demonstrated that the quality of the *G. pallida* genome presented in this paper allows for the mapping of complete effector families. With this resource, resequencing data from pathotypically diverse *G. pallida* populations will provide insight into the ecological diversification of this extreme range expander, and enable the development of a new pathotyping system that will facilitate the targeted and durable use of precious host plant resistances against this notorious plant pathogen.

## AUTHOR CONTRIBUTIONS

S.vdE. performed the DNA extraction and library preparations for PCNs. J.L. performed the DNA extraction and library preparations for *H. schachtii*. J.vS., P.T. and M.H. conceptualized the genome assembly pipeline. J.vS. and M.H. generated the genome assemblies. J.vS., M.S. and J.H. conceptualized the comparative genomics analyses. J.vS. performed the comparative genomics/effectoromics and phylogenetic analyses. J.vS. and J.H. wrote the manuscript. J.H. and G.S. acquired the main part of the funding and supervised the project. P.T., M.S., A.G., V.P. and G.S. substantially revised/commented on the manuscript. All author(s) read the final version of the manuscript and approved it.

## CONFLICT OF INTEREST

All authors declare that they have no conflict of interest.

### DATA AVAILIBILITY STATEMENT

Raw sequence reads have been deposited on the NCBI SRA under BioProject PRJEB2896 for *G. pallida* and under BioProject PRJNA767548 for *H. schachtii*. The genome assemblies of *G. pallida* (accession: GCA_020449905.1) and *H. schachtii* (accession: GCA_020449115.1) have been submitted to GenBank.
